# Effect of census-based correction of population figures on mortality rates in Germany

**DOI:** 10.1186/s12963-025-00361-5

**Published:** 2025-01-28

**Authors:** Andreas Stang, Markus Deckert

**Affiliations:** 1 Institute of Medical Informatics, Biometry and Epidemiology, University Hospital of Essen, Hufelandstr. 55, 45147 Essen, Germany; 2 School of Public Health, Department of Epidemiology, Boston University, 715 Albany Street, Talbot Building, Boston, MA 02118 USA; 3 Cancer Registry of North Rhine-Westphalia, Gesundheitscampus 10, 44801 Bochum, Germany

## Abstract

**Background:**

The population figures in Germany are obtained by updating the results of the latest census with information from the statistics on birth, deaths and migration statistics. The Census 2011 in Germany corrected population figures, which have only been updated over a long period of time. The aim of this work is to show the effect of the census-based correction of the population figures on the magnitude of mortality rates in Germany 2011–2013.

**Methods:**

We compared mortality rates (total, cancer, and cardiovascular disease) for the period 2011–2013 based on the uncorrected and Census 2011 corrected population figures. We also compared the effect of the choice of different standard populations in the age standardization of rates on the difference in uncorrected and corrected mortality rates.

**Results:**

There is a clear decline in age-specific cancer mortality among men aged 90 and over when using the uncorrected population figures, which is reversed as soon as the corrected population figures are used. Among women, there is hardly any difference between the uncorrected and corrected mortality rates. The correction of the population figures does not lead to a qualitatively different pattern in the mortality rates for cardiovascular diseases and myocardial infarction, but it increases the magnitude of the rates, particularly for elderly men. Standard populations with higher weights at older ages produced larger corrections in mortality rates.

**Conclusions:**

Even though the Census 2011 corrected nationwide mortality rates without age stratification differed only slightly from the uncorrected rates, there were noticeable increases in mortality, particularly in the city states of Hamburg and Berlin and in old age. Due to the particularly large error in the population figures in the older age range, an age standard that assigns lower weights at older ages should be used for age standardization of rates wherever possible.

**Supplementary Information:**

The online version contains supplementary material available at 10.1186/s12963-025-00361-5.

## Introduction

Population figures in Germany are obtained by updating the results of the latest census with information from the statistics on birth, deaths and migration statistics. Changes of nationality, other population corrections, and territorial changes are also included. A census is a complete collection of data on the population of a country on a specific date. Among other things, censuses provide the basis for the ongoing updating of the population figures. Censuses were carried out in the former Federal Republic of Germany in 1950, 1956 (building and housing census), 1961, 1970 and 1987, and in the former GDR in 1950, 1964, 1971 and 1981. Until 2011, the population figures for East and West Germany were updated on the basis of the 1981 and 1987 Census, respectively. East German population figures were extracted from the residents’ registration offices on the date of reunification (October 3, 1990) and updated further [1]. The 2011 census is the first all-German census since reunification (www.destatis.de, accessed Jan 3, 2024).

The Federal Bureau of Statistics produced population figures for the years 2011 (year of the Census) to 2013 using two methods: (1) updated population figures without accounting for the Census 2011 data and (2) updated population figures accounting for the Census 2011 data. A comparison of the two approaches for the year 2011 showed that the uncorrected population figures were too high without taking the 2011 Census into account. The overestimation of the population figures was greater for men than for women and was particularly high for those aged 90 and over. There was also a clear overestimation of the population figures for men aged 31–51 [1].

**Fig. 1:**
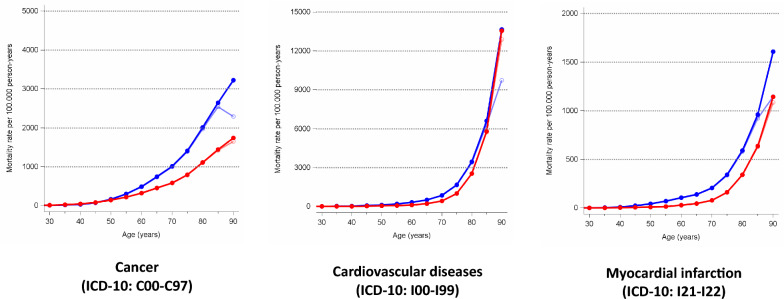
Age-mortality pattern of cancer, cardiovascular diseases, and myocardial infarction in Germany 2011–2013. Corrected rates (●), uncorrected rates (○); blue: men, red: women; all rates are crude rates; ICD-10: International Classification of Diseases, 10th edition

While the registration of births and deaths in Germany is of high quality [2], the registration of migrations is problematic. If migrations are not or incompletely registered, it can happen that people with more than one residence are counted more than once or that people who have left Germany are still counted as living in Germany. These errors lead to an inflation of the population figures [1]. Errors in the population figures have an influence on epidemiological measures such as the incidence and mortality of diseases. Interestingly, the effect of these errors on the level of Federal State specific and age-specific mortality rates in Germany has not yet been investigated.

The aim of this paper is to show the effect of the census-based correction of the population figures on the magnitude of mortality rates in Germany 2011–2013.

## Methoden

All population figures were provided electronically by the Federal Bureau of Statistics on August 28, 2015 upon request.

In the 2011 census, a new register-based method was used for the first time in Germany, in which existing register data was supplemented and corrected with the help of survey data. Around 10% of all people throughout Germany were randomly interviewed as part of household surveys. The Federal Bureau of Statistics calculated the population figures per Federal State, per age (1-year classes 0, 1, …,99, and ≥ 100 years), sex and calendar year (2011–2013). For the calendar years 2012 and 2013, the Federal Bureau of Statistics updated the population figures using the 2011 Census data. These population figures are referred to as "corrected" population figures.

In addition to calculating the population figures based on the 2011 census for the years 2011–2013, the Federal Bureau of Statistics also calculated the population data per Federal State, per age (1-year classes 0, 1, …89, and ≥ 90 years), sex and calendar year (2011–2013) based on the usual updating of population figures for the years 2011–2013, ignoring census data from 2011. This updated population data would have been used in Germany without the census 2011 for the years 2011–2013. These population figures are referred to as "*uncorrected*" population figures.

We downloaded the number of deaths (any cause), the number of cancer deaths (International Classification of Diseases, 10th edition, ICD-10 [3], C00-C97) by sex, age group (0–4, 5–9, 85–89, 90 + years) and calendar year (1998 through 2013) from the web page of the Federal Bureau of Statistics (www.destatis.de, accessed December 1, 2015). For the time 1998 through 2010, we used the official updated population figures as provided by the Federal Bureau of Statistics.

## Statistical methods

We calculated the absolute and relative changes in person-years for the entire period 2011–2013 per Federal State, sex and age group (0–4, 5–9, …, 85–89, ≥ 90 years). "The highest age group 90 years and more is an open-ended age group that in principle contains at least three 5-year age groups (90–94, 95–99 and 100 + years)." We calculated sex-specific total and cause-specific mortality rates overall and by age for specific causes of deaths of interest inclung cancer-related death (International Classification of Diseases, 10th edition, ICD-10 [3], C00-C97), cardiovascular deaths (I00-I99), deaths due to myocardial infarction (I21-I22), and for cancers deaths, which typically occur at an older age including prostate cancer (C61), kidney cancer (C64), and thyroid cancer (C73).

We also calculated age-standardized mortality rates. Mortality rates calculated by use of the uncorrected population figures were compared with mortality rates calculated by use of the corrected population figures for the period 2011–2013. Depending on the extent of the change in person-years in the individual age groups, the choice of age standard for age standardization of mortality rates can have more or less influence on the difference between uncorrected and corrected mortality rates. For this reason, the mortality rates were standardized using the European standard population, the World Standard Population and the Germany Standard Population of 2013 (according to the census) [4] (Suppl. Table 1). Differences of mortality rates based on uncorrected and corrected population figures were quantified on an absolute (rate difference) and relative scale (percentage difference of rates).

## Results

Based on the corrected population figures, the person-years of the population in Germany in 2011–2013 were 2.9 million person-years (by 2.4% in relative terms) lower for men and 1.6 million person-years (by 1.2% in relative terms) lower for women than based on the uncorrected population figures. The number of person-years was also lower in each of the 16 federal states for the corrected population figures. The reductions of person-years were particularly marked in the city states of Berlin (men: 5.7%; women: 4.1%) and Hamburg (men: 5.6%; women: 3.4%) (Table 1).

**Table 1:** Person-years of the calendar years 2011–2013 according to uncorrected and corrected population figures in the 16 Federal States of Germany

Federal State	Person-years (uncorrected, F)	Person-years (corrected, V)	Difference (V-F)	Relative Difference in %
*Men*				
BW	16,026,098	15,519,065	− 507,033	− 3.2
BY	18,674,937	18,361,104	− 313,833	− 1.7
BE	5,186,776	4,893,209	− 293,567	− 5.7
BB	3,708,110	3,620,840	− 87,270	− 2.4
HB	970,661	954,921	− 15,740	− 1.6
HH	2,658,289	2,509,411	− 148,878	− 5.6
HE	9,003,722	8,807,352	− 196,370	− 2.2
MV	2,421,944	2,370,852	− 51,092	− 2.1
NI	11,703,863	11,426,405	− 277,458	− 2.4
NW	26,172,495	25,604,542	− 567,953	− 2.2
RP	5,905,426	5,860,942	− 44,484	− 0.8
SL	1,479,084	1,452,359	− 26,725	− 1.8
SN	6,081,155	5,937,163	− 143,992	− 2.4
ST	3,389,562	3,327,970	− 61,592	− 1.8
SH	4,180,964	4,094,465	− 86,499	− 2.1
TH	3,283,653	3,212,748	− 70,905	− 2.2
Germany	120,846,739	117,953,348	− 2,893,391	− 2.4
*Women*				
BW	16,426,339	16,117,543	− 308,796	− 1.9
BY	19,224,645	19,095,841	− 128,804	− 0.7
BE	5,379,243	5,158,279	− 220,964	− 4.1
BB	3,774,196	3,737,235	− 36,961	− 1.0
HB	1,015,061	1,006,621	− 8,440	− 0.8
HH	2,764,375	2,669,240	− 95,135	− 3.4
HE	9,303,431	9,210,497	− 92,934	− 1.0
MV	2,471,554	2,442,176	− 29,378	− 1.2
NI	12,052,841	11,911,709	− 141,132	− 1.2
NW	27,355,106	27,053,416	− 301,690	− 1.1
RP	6,091,865	6,113,821	21,956	0.4
SL	1,554,564	1,536,243	− 18,321	− 1.2
SN	6,317,051	6,223,722	− 93,329	− 1.5
ST	3,523,322	3,479,313	− 44,009	− 1.2
SH	4,337,999	4,322,547	− 15,452	− 0.4
TH	3,361,700	3,317,583	− 44,117	− 1.3
Germany	124,953,292	123,395,786	− 1,557,506	− 1.2

BW, Baden-Wuerttemberg; BY, Bavaria; BE, Berlin; BB, Brandenburg; HB, Bremen; HH, Hamburg; HE, Hessia; MV, Mecklenburg-West Pomerania; NI, Lower Saxony; NW, North Rhine-Westphalia; RP, Rhineland-Palatinate; SL, Saarland; SN, Saxony; ST, Saxony-Anhalt; SH, Schleswig–Holstein; TH, Thuringia

The age-specific analysis of person-years shows that the relative reduction in person-years was greatest for people aged 90 and over (men: 28.9%, women: 5.0%). There were also noticeable corrections to the population figures in middle age (20–64 years) (Germany-wide, men: 2.1–3.9%, women: 0.8–2.3%) (Table 2). Within the city states, there were even more marked corrections to the population figures (Hamburg: men, 20–64 years: 2.4–11.7%; women 1.9–6.7%; Berlin: men: 4.6–10.0%, women: 4.0–6.9%) (Suppl. Figure 1).

**Table 2:** Age and sex-specific differences in the person-years of the uncorrected and corrected population figures for the years 2011–2013 in 16 Federal States of Germany

5-year age groups	Person-years (uncorrected, F)	Person− years (corrected, V)	Delta (V-F)	Relative change %
*Men*				
0	5,247,928	5,191,618	− 56,310	− 1.1
5	5,413,551	5,374,739	− 38,812	− 0.7
10	5,946,641	5,932,884	− 13,757	− 0.2
15	6,292,906	6,195,843	− 97,063	− 1.5
20	7,538,110	7,323,217	− 214,893	− 2.9
25	7,714,739	7,473,640	− 241,099	− 3.1
30	7,598,549	7,371,716	− 226,833	− 3.0
35	7,302,601	7,038,419	− 264,182	− 3.6
40	9,458,595	9,085,694	− 372,901	− 3.9
45	10,899,212	10,607,183	− 292,029	− 2.7
50	9,887,309	9,680,556	− 206,753	− 2.1
55	8,361,279	8,184,829	− 176,450	− 2.1
60	7,286,324	7,132,221	− 154,103	− 2.1
65	5,877,322	5,765,995	− 111,327	− 1.9
70	6,827,953	6,734,174	− 93,779	− 1.4
75	4,672,129	4,602,213	− 69,916	− 1.5
80	2,740,839	2,683,320	− 57,519	− 2.1
85	1,241,988	1,191,759	− 50,229	− 4.0
90 +	538,764	383,328	− 155,436	− 28.9
*Women*				
0	4,986,616	4,926,910	− 59,706	− 1.2
5	5,136,665	5,094,425	− 42,240	− 0.8
10	5,646,572	5,631,578	− 14,994	− 0.3
15	5,959,082	5,881,266	− 77,816	− 1.3
20	7,188,050	7,022,268	− 165,782	− 2.3
25	7,394,206	7,267,069	− 127,137	− 1.7
30	7,372,119	7,277,492	− 94,627	− 1.3
35	7,093,033	6,977,093	− 115,940	− 1.6
40	9,081,501	8,910,894	− 170,607	− 1.9
45	10,430,055	10,325,678	− 104,377	− 1.0
50	9,666,761	9,585,629	− 81,132	− 0.8
55	8,452,973	8,379,827	− 73,146	− 0.9
60	7,577,373	7,490,634	− 86,739	− 1.1
65	6,332,878	6,257,909	− 74,969	− 1.2
70	7,865,154	7,801,632	− 63,522	− 0.8
75	6,020,628	5,967,026	− 53,602	− 0.9
80	4,328,218	4,285,736	− 42,482	− 1.0
85	2,924,389	2,890,804	− 33,585	− 1.1
90 +	1,497,019	1,421,916	− 75,103	− 5.0

BW, Baden-Wuerttemberg, BY, Bavaria; BE, Berlin; BB, Brandenburg; HB, Bremen; HH, Hamburg; HE, Hessia; MV, Mecklenburg-West Pomerania; NI, Lower Saxony; NW, North Rhine-Westphalia; RP, Rhineland-Palatinate; SL, Saarland; SN, Saxony; ST, Saxony-Anhalt; SH, Schleswig–Holstein; TH, Thuringia

There is a clear decline in age-specific cancer mortality among men aged 90 and over when using the uncorrected population figures, which is reversed as soon as the corrected population figures are used. Among women, there is hardly any difference between the uncorrected and corrected mortality rates. The correction of the population figures does not lead to a qualitatively different pattern in the mortality rates for cardiovascular diseases and myocardial infarction, but it does increase the magnitude of the rates, particularly for elderly men (Fig. 1).

**Fig. 2:**
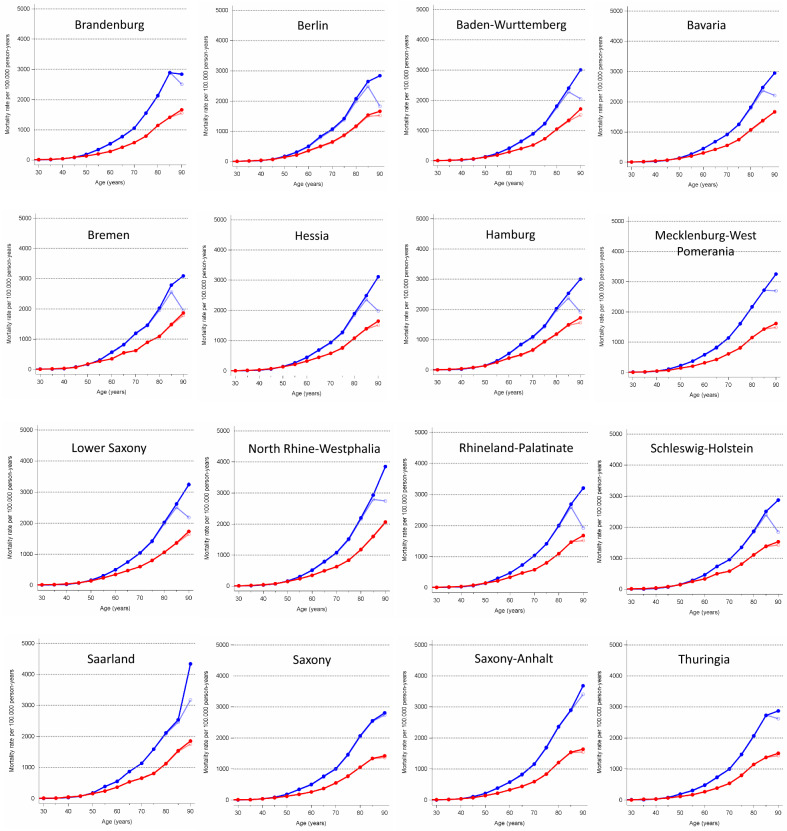
Age-specific cancer mortality rates per 100,000 person-years based on corrected (●) and uncorrected population figures (○) in the 16 Federal States of Germany, 2011–2013. Rates based on corrected population figures (●), rates based on uncorrected population figures (○); blue: men, red: women; ICD-10: International Classification of Diseases, 10th edition

On the basis of the uncorrected population figures, a decline in age-specific cancer mortality at the highest age (≥ 90 years) is also observed for men in most federal states with the exception of Saarland, Saxony, and Saxony-Anhalt. Using the corrected data, however, a strictly monotonic increase in age-specific cancer mortality rates can be seen for both men and women, with the exception of Brandenburg (Fig. 2). The nationwide time trend (1998–2013) in age-standardized mortality for cancer, cardiovascular diseases and heart attacks remain virtually unchanged after correction of the population figures (data not shown).

The difference between the uncorrected and corrected age-standardized mortality rates for cancer, cardiovascular disease and myocardial infarction depends on which standard population is used. Standard populations with higher weights at older ages (e.g. German standard, 2013) produce larger corrections in mortality rates than standard populations with low weights at older ages (e.g. world standard population) (Suppl. Tables 1, 2, 3, 4, 5 and 6).

The correction of the population figures by the Census 2011 is particularly noticeable for diseases with a high average age of death. For example, the mortality rate for prostate cancer in men aged 90 years in Germany and older is underestimated by 247 per 100,000 person-years using the uncorrected population figures. For less common cancers such as kidney and thyroid cancer, this rate is underestimated by 22.4 per 100,000 person-years and 2.0 per 100,000 person-years, respectively (Suppl. Figure 2).

## Discussion

We found that the relative changes of mortality rates after correction of population figures were greater for men than for women and occur particularly in the oldest population segment (90 + years). Mortality rates increased after correction, particularly in the city states of Hamburg and Berlin, where the largest population correction were made. It is known that one of the sources of error in the uncorrected population figures was that people who had already died continued to be counted as still alive. This error increases with increasing age due to increasing mortality. Due to the last open age group "90 years and more", which in principle includes several 5-year age groups, there is a particularly marked correction effect of person-years by the census. Mortality rates of diseases that occur mainly in old age are particularly affected by the correction of population figures. The relative mortality rate increases after correction of population figures in age-standardized rates get larger the more the standard population gives weight to older age. The comparison of mortality rates between sexes based on uncorrected statistics is potentially biased as there are greater differences between uncorrected and corrected data for men than for women.

In contrast to our results, the corrected mortality rates for the 15 leading causes of death in the USA based on the Census 2000 became lower. However, age-specific mortality rates became higher for children and the very elderly [5]. The correction of the population figures with the census 2011 in Germany also has an influence on the life expectancy determined from the mortality tables. A comparison of life expectancy based on the uncorrected and corrected population figures in two federal states of Germany showed that life expectancy is slightly overestimated in the majority of districts (i.e. by a median of up to 2 months) [6].

An old age decline in cancer mortality rates has been observed in several countries. Based on detailed analyses of cancer incidence or cancer mortality in old age, various authors have generated hypotheses in the past that could explain this phenomenon: (1) cancer-susceptible people have usually already developed cancer before reaching old age so that there are hardly any people with cancer susceptibility among the remaining elderly [7–9], (2) older people can remain asymptomatic in case of illness, so that the disease is not diagnosed, (3) the intensity of diagnostic work-up is generally lower in older age [10, 11]. Our study as well as other studies [5, 12, 13] show that declines in cancer-specific or cardiovascular-specific mortality in old age can very easily occur due to errors in the extrapolation of population figures between Census surveys.

In view of the already existing susceptibility to error of the unicausal cause of death statistics in Germany [14, 15], particularly in old age, a further source of error, namely incorrect population figures, should be minimized as far as possible, as otherwise an interpretation of disease-specific mortality in old age is even more problematic.

Even though the Census 2011 corrected Germany-wide mortality rates without age stratification differed only slightly from the uncorrected rates, there were noticeable increases in mortality, particularly in the city states of Hamburg and Berlin and in old age. Due to the particularly large error in the population figures in the older age range, an age standard that assigns lower weights at older ages should be used for age standardization of rates wherever possible. A regular census-based correction of the updated population figures is important for epidemiological measures in Germany.

## Supplementary Information

Additional file 1.

## Data Availability

Included in the manuscript
