# Supplementary material for: Effect of census-based correction of population figures on mortality rates in Germany
Source: Popul Health Metr. 2025 Jan 28;23:1. doi: 10.1186/s12963-025-00361-5 (PMC11773962; doi:10.1186/s12963-025-00361-5)
Supplement: Supplementary file 1 — Additional file 1. [file 12963_2025_361_MOESM1_ESM.docx]

Online Supplement

**Suppl. Table 1 Statistical weights from standard populations**

| **5-year age groups** | **World Standard Population** | **European Standard Population** | **Germany 2013 Standard** |
| --- | --- | --- | --- |
| 0 | 12000 | 8000 | 4185 |
| 5 | 10000 | 7000 | 4297 |
| 10 | 9000 | 7000 | 4697 |
| 15 | 9000 | 7000 | 4985 |
| 20 | 8000 | 7000 | 5845 |
| 25 | 8000 | 7000 | 6190 |
| 30 | 6000 | 7000 | 6197 |
| 35 | 6000 | 7000 | 5762 |
| 40 | 6000 | 7000 | 7038 |
| 45 | 6000 | 7000 | 8614 |
| 50 | 5000 | 7000 | 8203 |
| 55 | 4000 | 6000 | 6977 |
| 60 | 4000 | 5000 | 6230 |
| 65 | 3000 | 4000 | 4858 |
| 70 | 2000 | 3000 | 5878 |
| 75 | 1000 | 2000 | 4662 |
| 80 | 500 | 1000 | 2867 |
| 85+ | 500 | 1000 | 2514 |
|  |  |  |  |
| Total | 100000 | 100000 | 100000 |

World Standard and European Standard Population weights from [4].

**Suppl. Table 2 Cancer mortality rates (C00-C97) per 100,000 person-years based on the uncorrected and corrected population figures for 2011-2013, men**

|  |  |  | | | |  | **Age-standardized rates** | | | | | | | | | | | | | |
| --- | --- | --- | --- | --- | --- | --- | --- | --- | --- | --- | --- | --- | --- | --- | --- | --- | --- | --- | --- | --- |
|  | **Deaths** | **Crude rates** | | | |  | **World Standard Population** | | | |  | **European Standarad Population** | | | |  | **Germany 2013 Population** | | | |
|  | **N** | **F** | **V** | **Abs. Diff.** | **Rel. Diff. %** |  | **F** | **V** | **Abs. Diff.** | **Rel. Diff. %** |  | **F** | **V** | **Abs. Diff.** | **Rel. Diff. %** |  | **F** | **V** | **Abs. Diff.** | **Rel. Diff. %** |
| BW | 40.060 | 250,0 | 258,1 | 8,1 | 3,3 |  | 107,2 | 112,1 | 4,9 | 4,9 |  | 166,1 | 174,2 | 8,1 | 4,9 |  | 304,0 | 320,3 | 16,3 | 5,4 |
| BY | 49.014 | 262,5 | 266,9 | 4,4 | 1,7 |  | 114,8 | 117,4 | 2,6 | 2,6 |  | 177,0 | 181,6 | 4,6 | 2,6 |  | 320,8 | 331,0 | 10,2 | 3,2 |
| BE | 14.033 | 270,6 | 286,8 | 16,2 | 6,0 |  | 128,0 | 135,7 | 7,7 | 7,7 |  | 196,5 | 208,9 | 12,4 | 6,3 |  | 352,8 | 376,8 | 24,0 | 6,8 |
| BB | 12.915 | 348,3 | 356,7 | 8,4 | 2,4 |  | 138,0 | 140,5 | 2,5 | 2,5 |  | 212,9 | 216,7 | 3,8 | 1,8 |  | 385,5 | 391,7 | 6,2 | 1,6 |
| HB | 3.150 | 324,5 | 329,9 | 5,4 | 1,6 |  | 135,5 | 141,0 | 5,5 | 5,5 |  | 207,4 | 216,9 | 9,5 | 4,6 |  | 371,1 | 391,6 | 20,5 | 5,5 |
| HH | 7.182 | 270,2 | 286,2 | 16,0 | 5,9 |  | 128,8 | 135,1 | 6,3 | 6,3 |  | 197,6 | 207,9 | 10,3 | 5,2 |  | 355,3 | 375,6 | 20,3 | 5,7 |
| HE | 24.709 | 274,4 | 280,6 | 6,2 | 2,2 |  | 115,0 | 119,5 | 4,5 | 4,5 |  | 177,2 | 185,1 | 7,9 | 4,4 |  | 321,2 | 337,9 | 16,7 | 5,2 |
| MV | 8.794 | 363,1 | 370,9 | 7,8 | 2,2 |  | 148,1 | 151,3 | 3,2 | 3,2 |  | 226,9 | 231,7 | 4,8 | 2,1 |  | 404,9 | 412,9 | 8,0 | 2,0 |
| NI | 36.151 | 308,9 | 316,4 | 7,5 | 2,4 |  | 127,0 | 130,8 | 3,8 | 3,8 |  | 195,9 | 202,4 | 6,5 | 3,3 |  | 354,2 | 368,2 | 14,0 | 4,0 |
| NW | 83.183 | 317,8 | 324,9 | 7,1 | 2,2 |  | 133,1 | 137,1 | 4,0 | 4,0 |  | 206,3 | 213,2 | 6,9 | 3,3 |  | 377,4 | 392,0 | 14,6 | 3,9 |
| RP | 18.237 | 308,8 | 311,2 | 2,4 | 0,8 |  | 124,7 | 127,4 | 2,7 | 2,7 |  | 192,6 | 197,8 | 5,2 | 2,7 |  | 349,8 | 362,4 | 12,6 | 3,6 |
| SL | 5.433 | 367,3 | 374,1 | 6,8 | 1,8 |  | 143,5 | 145,7 | 2,2 | 2,2 |  | 221,0 | 224,9 | 3,9 | 1,8 |  | 396,0 | 404,7 | 8,7 | 2,2 |
| SN | 21.340 | 350,9 | 359,4 | 8,5 | 2,4 |  | 133,0 | 135,9 | 2,9 | 2,9 |  | 204,6 | 208,7 | 4,1 | 2,0 |  | 367,6 | 374,2 | 6,6 | 1,8 |
| ST | 13.535 | 399,3 | 406,7 | 7,4 | 1,9 |  | 152,0 | 154,4 | 2,4 | 2,4 |  | 234,1 | 237,8 | 3,7 | 1,6 |  | 422,3 | 428,8 | 6,5 | 1,5 |
| SH | 12.604 | 301,5 | 307,8 | 6,3 | 2,1 |  | 120,9 | 123,3 | 2,4 | 2,4 |  | 186,2 | 190,7 | 4,5 | 2,4 |  | 335,2 | 346,3 | 11,1 | 3,3 |
| TH | 10.946 | 333,3 | 340,7 | 7,4 | 2,2 |  | 130,3 | 132,8 | 2,5 | 2,5 |  | 201,4 | 205,0 | 3,6 | 1,8 |  | 365,7 | 371,6 | 5,9 | 1,6 |
| Germany | 361.286 | 299,0 | 306,3 | 7,3 | 2,5 |  | 125,1 | 128,9 | 3,8 | 3,1 |  | 193,1 | 199,5 | 6,4 | 3,3 |  | 350,2 | 363,7 | 13,5 | 3,8 |

Legend: F: Rates based on uncorrected population figures; V: Rates based on corrected population data; Abs. diff: Rate difference; Rel. diff.: relative percentage change in rates;

**Suppl. Table 3 Cancer mortality rates (C00-C97) per 100,000 person-years based on the uncorrected and corrected population figures for 2011-2013, women**

|  |  |  | | | |  | **Age-standardized rates** | | | | | | | | | | | | | |
| --- | --- | --- | --- | --- | --- | --- | --- | --- | --- | --- | --- | --- | --- | --- | --- | --- | --- | --- | --- | --- |
|  | **Deaths** | **Crude rates** | | | |  | **World Standard Population** | | | |  | **European Standarad Population** | | | |  | **Germany 2013 Population** | | | |
|  | **N** | **F** | **V** | **Abs. Diff.** | **Rel. Diff. %** |  | **F** | **V** | **Abs. Diff.** | **Rel. Diff. %** |  | **F** | **V** | **Abs. Diff.** | **Rel. Diff. %** |  | **F** | **V** | **Abs. Diff.** | **Rel. Diff. %** |
| BW | 34.398 | 209,4 | 213,4 | 4,0 | 1,9 |  | 74,5 | 76,0 | 1,5 | 2,0 |  | 112,7 | 115,1 | 2,4 | 2,1 |  | 197,4 | 201,8 | 4,4 | 2,2 |
| BY | 42.721 | 222,2 | 223,7 | 1,5 | 0,7 |  | 79,5 | 80,1 | 0,6 | 0,7 |  | 120,0 | 120,9 | 0,9 | 0,7 |  | 209,1 | 210,8 | 1,7 | 0,8 |
| BE | 12.414 | 230,8 | 240,7 | 9,9 | 4,3 |  | 87,4 | 90,9 | 3,5 | 4,0 |  | 132,0 | 137,2 | 5,2 | 3,9 |  | 230,0 | 238,5 | 8,5 | 3,7 |
| BB | 9.835 | 260,6 | 263,2 | 2,6 | 1,0 |  | 82,6 | 83,4 | 0,8 | 0,9 |  | 124,4 | 125,5 | 1,1 | 0,9 |  | 216,7 | 218,8 | 2,1 | 1,0 |
| HB | 2.802 | 276,0 | 278,4 | 2,4 | 0,8 |  | 94,4 | 95,7 | 1,3 | 1,4 |  | 142,0 | 143,9 | 1,9 | 1,4 |  | 241,9 | 245,2 | 3,3 | 1,4 |
| HH | 6.697 | 242,3 | 250,9 | 8,6 | 3,6 |  | 92,9 | 96,0 | 3,1 | 3,3 |  | 139,8 | 144,4 | 4,6 | 3,2 |  | 241,3 | 248,8 | 7,5 | 3,1 |
| HE | 21.509 | 231,2 | 233,5 | 2,3 | 1,0 |  | 82,8 | 84,0 | 1,2 | 1,4 |  | 124,4 | 126,3 | 1,9 | 1,5 |  | 214,8 | 218,3 | 3,5 | 1,6 |
| MV | 6.632 | 268,3 | 271,6 | 3,3 | 1,2 |  | 84,5 | 85,5 | 1,0 | 1,3 |  | 127,4 | 129,0 | 1,6 | 1,3 |  | 221,7 | 224,5 | 2,8 | 1,3 |
| NI | 29.957 | 248,5 | 251,5 | 3,0 | 1,2 |  | 86,9 | 87,8 | 0,9 | 1,0 |  | 130,2 | 131,6 | 1,4 | 1,0 |  | 223,1 | 225,5 | 2,4 | 1,1 |
| NW | 71.583 | 261,7 | 264,6 | 2,9 | 1,1 |  | 90,6 | 91,4 | 0,8 | 0,9 |  | 136,7 | 137,9 | 1,2 | 0,9 |  | 237,7 | 239,6 | 1,9 | 0,8 |
| RP | 15.280 | 250,8 | 249,9 | -0,9 | -0,4 |  | 86,4 | 86,1 | -0,3 | -0,3 |  | 129,8 | 129,6 | -0,2 | -0,2 |  | 223,5 | 223,8 | 0,3 | 0,1 |
| SL | 4.469 | 287,5 | 290,9 | 3,4 | 1,2 |  | 94,2 | 95,0 | 0,8 | 0,9 |  | 140,6 | 141,9 | 1,3 | 0,9 |  | 239,7 | 242,4 | 2,7 | 1,1 |
| SN | 16.758 | 265,3 | 269,3 | 4,0 | 1,5 |  | 75,9 | 76,8 | 0,9 | 1,2 |  | 114,8 | 116,1 | 1,3 | 1,1 |  | 201,3 | 203,4 | 2,1 | 1,0 |
| ST | 10.502 | 298,1 | 301,8 | 3,7 | 1,3 |  | 86,6 | 87,6 | 1,0 | 1,1 |  | 130,9 | 132,4 | 1,5 | 1,1 |  | 228,1 | 230,7 | 2,6 | 1,1 |
| SH | 11.031 | 254,3 | 255,2 | 0,9 | 0,4 |  | 88,9 | 88,9 | 0,0 | 0,0 |  | 133,0 | 133,2 | 0,2 | 0,2 |  | 226,5 | 227,5 | 1,0 | 0,5 |
| TH | 8.674 | 258,0 | 261,5 | 3,5 | 1,3 |  | 75,9 | 76,8 | 0,9 | 1,2 |  | 115,3 | 116,6 | 1,3 | 1,1 |  | 204,3 | 206,4 | 2,1 | 1,0 |
| Germany | 305.262 | 244,3 | 247,4 | 3,1 | 1,3 |  | 83,9 | 84,9 | 1,0 | 1,2 |  | 126,5 | 128,0 | 1,5 | 1,2 |  | 219,6 | 222,3 | 2,7 | 1,3 |

Legend: F: Rates based on uncorrected population figures; V: Rates based on corrected population data; Abs. diff: Rate difference; Rel. diff.: relative percentage change in rates;

**Suppl. Table 4 Cardiovascular mortality rates (I00-I99) per 100,000 person-years based on the uncorrected and corrected population figures for 2011-2013, men**

|  |  |  | | | |  | **Age-standardized rates** | | | | | | | | | | | | | |
| --- | --- | --- | --- | --- | --- | --- | --- | --- | --- | --- | --- | --- | --- | --- | --- | --- | --- | --- | --- | --- |
|  | **Deaths** | **Crude rates** | | | |  | **World Standard Population** | | | |  | **European Standarad Population** | | | |  | **Germany 2013 Population** | | | |
|  | **N** | **F** | **V** | **Abs. Diff.** | **Rel. Diff. %** |  | **F** | **V** | **Abs. Diff.** | **Rel. Diff. %** |  | **F** | **V** | **Abs. Diff.** | **Rel. Diff. %** |  | **F** | **V** | **Abs. Diff.** | **Rel. Diff. %** |
| BW | 51,602 | 322.0 | 332.5 | 10.5 | 3.3 |  | 118.0 | 126.0 | 8.0 | 6.8 |  | 201.2 | 216.0 | 14.8 | 7.4 |  | 430.1 | 464.6 | 34.5 | 8.0 |
| BY | 66,813 | 357.8 | 363.9 | 6.1 | 1.7 |  | 134.1 | 139.8 | 5.7 | 4.3 |  | 228.4 | 239.4 | 11.0 | 4.8 |  | 486.0 | 512.8 | 26.8 | 5.5 |
| BE | 13,646 | 263.1 | 278.9 | 15.8 | 6.0 |  | 116.5 | 126.2 | 9.7 | 8.3 |  | 193.4 | 210.7 | 17.3 | 8.9 |  | 396.7 | 435.7 | 39.0 | 9.8 |
| BB | 15,496 | 417.9 | 428.0 | 10.1 | 2.4 |  | 154.8 | 157.7 | 2.9 | 1.9 |  | 262.0 | 266.9 | 4.9 | 1.9 |  | 552.9 | 562.6 | 9.7 | 1.8 |
| HB | 3911 | 402.9 | 409.6 | 6.7 | 1.7 |  | 147.8 | 157.8 | 10.0 | 6.8 |  | 242.9 | 261.7 | 18.8 | 7.7 |  | 491.2 | 535.9 | 44.7 | 9.1 |
| HH | 7540 | 283.6 | 300.5 | 16.9 | 6.0 |  | 114.9 | 123.8 | 8.9 | 7.7 |  | 193.6 | 209.9 | 16.3 | 8.4 |  | 406.2 | 443.7 | 37.5 | 9.2 |
| HE | 31,472 | 349.5 | 357.3 | 7.8 | 2.2 |  | 122.5 | 130.9 | 8.4 | 6.9 |  | 208.8 | 224.8 | 16.0 | 7.7 |  | 445.6 | 483.8 | 38.2 | 8.6 |
| MV | 10,289 | 424.8 | 434.0 | 9.2 | 2.2 |  | 163.6 | 167.7 | 4.1 | 2.5 |  | 273.5 | 280.4 | 6.9 | 2.5 |  | 566.2 | 580.4 | 14.2 | 2.5 |
| NI | 47,077 | 402.2 | 412.0 | 9.8 | 2.4 |  | 141.6 | 149.2 | 7.6 | 5.4 |  | 240.2 | 254.6 | 14.4 | 6.0 |  | 508.4 | 543.0 | 34.6 | 6.8 |
| NW | 92,659 | 354.0 | 361.9 | 7.9 | 2.2 |  | 129.4 | 135.6 | 6.2 | 4.8 |  | 218.5 | 230.1 | 11.6 | 5.3 |  | 460.0 | 487.6 | 27.6 | 6.0 |
| RP | 23,780 | 402.7 | 405.7 | 3.0 | 0.7 |  | 138.0 | 145.3 | 7.3 | 5.3 |  | 233.5 | 247.8 | 14.3 | 6.1 |  | 492.7 | 528.6 | 35.9 | 7.3 |
| SL | 6347 | 429.1 | 437.0 | 7.9 | 1.8 |  | 145.1 | 149.8 | 4.7 | 3.2 |  | 243.7 | 252.6 | 8.9 | 3.7 |  | 509.7 | 531.3 | 21.6 | 4.2 |
| SN | 29,342 | 482.5 | 494.2 | 11.7 | 2.4 |  | 160.7 | 163.7 | 3.0 | 1.9 |  | 273.0 | 277.8 | 4.8 | 1.8 |  | 577.6 | 586.8 | 9.2 | 1.6 |
| ST | 17,563 | 518.6 | 527.7 | 9.1 | 1.8 |  | 180.6 | 183.7 | 3.1 | 1.7 |  | 305.0 | 310.2 | 5.2 | 1.7 |  | 640.7 | 651.8 | 11.1 | 1.7 |
| SH | 16,817 | 402.2 | 410.7 | 8.5 | 2.1 |  | 141.0 | 148.1 | 7.1 | 5.0 |  | 237.3 | 251.3 | 14.0 | 5.9 |  | 497.4 | 532.5 | 35.1 | 7.1 |
| TH | 14,595 | 444.5 | 454.3 | 9.8 | 2.2 |  | 158.6 | 161.5 | 2.9 | 1.8 |  | 268.9 | 273.5 | 4.6 | 1.7 |  | 569.0 | 578.0 | 9.0 | 1.6 |
| Germany | 448,949 | 371.5 | 380.6 | 9.1 | 2.4 |  | 135.3 | 142.0 | 6.7 | 5.0 |  | 229.0 | 241.6 | 12.6 | 5.5 |  | 483.6 | 513.3 | 29.7 | 6.1 |

Legend: F: Rates based on uncorrected population figures; V: Rates based on corrected population data; Abs. diff: Rate difference; Rel. diff.: relative percentage change in rates;

**Suppl. Table 5 Cardiovascular mortality rates (I00-I99) per 100,000 person-years based on the uncorrected and corrected population figures for 2011-2013, women**

|  |  |  | | | |  |  | **Age-standardized rates** | | | | | | | | | | | | | |
| --- | --- | --- | --- | --- | --- | --- | --- | --- | --- | --- | --- | --- | --- | --- | --- | --- | --- | --- | --- | --- | --- |
|  | **Deaths** | **Crude rates** | | | |  |  | **World Standard Population** | | | |  | **European Standarad Population** | | | |  | **Germany 2013 Population** | | | |
|  | **N** | **F** | **V** | **Abs. Diff.** | **Rel. Diff. %** |  |  | **F** | **V** | **Abs. Diff.** | **Rel. Diff. %** |  | **F** | **V** | **Abs. Diff.** | **Rel. Diff. %** |  | **F** | **V** | **Abs. Diff.** | **Rel. Diff. %** |
| BW | 67,740 | 412.4 | 420.3 | 7.9 | 1.9 |  |  | 78.9 | 81.6 | 2.7 | 3.4 |  | 144.0 | 149.2 | 5.2 | 3.6 |  | 335.8 | 348.4 | 12.6 | 3.8 |
| BY | 88,086 | 458.2 | 461.3 | 3.1 | 0.7 |  |  | 89.0 | 89.8 | 0.8 | 0.9 |  | 162.4 | 163.9 | 1.5 | 0.9 |  | 378.1 | 381.9 | 3.8 | 1.0 |
| BE | 18,813 | 349.7 | 364.7 | 15 | 4.3 |  |  | 78.7 | 81.9 | 3.2 | 4.1 |  | 141.5 | 147.3 | 5.8 | 4.1 |  | 323.3 | 336.5 | 13.2 | 4.1 |
| BB | 20,320 | 538.4 | 543.7 | 5.3 | 1.0 |  |  | 100.1 | 101.6 | 1.5 | 1.5 |  | 182.4 | 185.2 | 2.8 | 1.5 |  | 423.6 | 430.4 | 6.8 | 1.6 |
| HB | 5080 | 500.5 | 504.7 | 4.2 | 0.8 |  |  | 93.6 | 95.2 | 1.6 | 1.7 |  | 165.3 | 168.1 | 2.8 | 1.7 |  | 371.0 | 377.3 | 6.3 | 1.7 |
| HH | 10,582 | 382.8 | 396.4 | 13.6 | 3.6 |  |  | 77.0 | 79.7 | 2.7 | 3.5 |  | 139.0 | 143.9 | 4.9 | 3.5 |  | 319.7 | 330.8 | 11.1 | 3.5 |
| HE | 41,150 | 442.3 | 446.8 | 4.5 | 1.0 |  |  | 84.2 | 86.2 | 2.0 | 2.4 |  | 153.0 | 156.8 | 3.8 | 2.5 |  | 354.5 | 363.6 | 9.1 | 2.6 |
| MV | 13,202 | 534.2 | 540.6 | 6.4 | 1.2 |  |  | 101.1 | 102.9 | 1.8 | 1.8 |  | 182.3 | 185.6 | 3.3 | 1.8 |  | 419.8 | 427.8 | 8.0 | 1.9 |
| NI | 60,804 | 504.5 | 510.5 | 6.0 | 1.2 |  |  | 93.2 | 94.7 | 1.5 | 1.6 |  | 168.3 | 171.1 | 2.8 | 1.7 |  | 387.9 | 394.5 | 6.6 | 1.7 |
| NW | 124,179 | 454.0 | 459.0 | 5.0 | 1.1 |  |  | 88.2 | 89.0 | 0.8 | 0.9 |  | 158.7 | 160.0 | 1.3 | 0.8 |  | 363.2 | 366.2 | 3.0 | 0.8 |
| RP | 31,129 | 511.0 | 509.2 | -1.8 | -0.4 |  |  | 93.4 | 94.4 | 1.0 | 1.1 |  | 168.7 | 170.9 | 2.2 | 1.3 |  | 388.2 | 394.0 | 5.8 | 1.5 |
| SL | 8071 | 519.2 | 525.4 | 6.2 | 1.2 |  |  | 92.9 | 94.5 | 1.6 | 1.7 |  | 165.6 | 168.7 | 3.1 | 1.9 |  | 375.1 | 382.7 | 7.6 | 2.0 |
| SN | 41,967 | 664.3 | 674.3 | 10.0 | 1.5 |  |  | 99.2 | 100.4 | 1.2 | 1.2 |  | 181.4 | 183.8 | 2.4 | 1.3 |  | 423.9 | 429.4 | 5.5 | 1.3 |
| ST | 23,698 | 672.6 | 681.1 | 8.5 | 1.3 |  |  | 110.6 | 112.3 | 1.7 | 1.5 |  | 200.3 | 203.4 | 3.1 | 1.5 |  | 462.4 | 469.6 | 7.2 | 1.6 |
| SH | 22,111 | 509.7 | 511.5 | 1.8 | 0.4 |  |  | 95.8 | 97.3 | 1.5 | 1.6 |  | 172.3 | 175.5 | 3.2 | 1.9 |  | 395.9 | 403.9 | 8.0 | 2.0 |
| TH | 19,945 | 593.3 | 601.1 | 7.8 | 1.3 |  |  | 100.7 | 102.0 | 1.3 | 1.3 |  | 183.2 | 185.7 | 2.5 | 1.4 |  | 425.2 | 431.0 | 5.8 | 1.4 |
| Germany | 596,877 | 477.7 | 483.7 | 6.0 | 1.3 |  |  | 89.8 | 91.3 | 1.5 | 1.7 |  | 162.7 | 165.6 | 2.9 | 1.8 |  | 376.0 | 382.7 | 6.7 | 1.8 |

Legend: F: Rates based on uncorrected population figures; V: Rates based on corrected population data; Abs. diff: Rate difference; Rel. diff.: relative percentage change in rates;

**Suppl. Table 6 Myocardial infarction mortality rates (I21-I22) per 100,000 person-years based on the uncorrected and corrected population figures for 2011-2013, men**

|  |  |  | | | |  | **Age-standardized rates** | | | | | | | | | | | | | |
| --- | --- | --- | --- | --- | --- | --- | --- | --- | --- | --- | --- | --- | --- | --- | --- | --- | --- | --- | --- | --- |
|  | **Deaths** | **Crude rates** | | | |  | **World Standard Population** | | | |  | **European Standarad Population** | | | |  | **Germany 2013 Population** | | | |
|  | **N** | **F** | **V** | **Abs. Diff.** | **Rel. Diff. %** |  | **F** | **V** | **Abs. Diff.** | **Rel. Diff. %** |  | **F** | **V** | **Abs. Diff.** | **Rel. Diff. %** |  | **F** | **V** | **Abs. Diff.** | **Rel. Diff. %** |
| BW | 10.701 | 66,8 | 69,0 | 2,2 | 3,3 |  | 27,2 | 28,7 | 1,5 | 5,4 |  | 43,6 | 46,2 | 2,6 | 5,9 |  | 84,2 | 89,7 | 5,5 | 6,5 |
| BY | 13.759 | 73,7 | 74,9 | 1,2 | 1,7 |  | 30,7 | 31,6 | 0,9 | 3,0 |  | 49,0 | 50,7 | 1,7 | 3,4 |  | 93,7 | 97,6 | 3,9 | 4,2 |
| BE | 3.135 | 60,4 | 64,1 | 3,7 | 6,0 |  | 28,1 | 30,1 | 2,0 | 7,1 |  | 44,5 | 47,9 | 3,4 | 7,5 |  | 84,0 | 90,9 | 6,9 | 8,2 |
| BB | 4.245 | 114,5 | 117,2 | 2,7 | 2,4 |  | 44,1 | 45,0 | 0,9 | 1,9 |  | 70,9 | 72,2 | 1,3 | 1,8 |  | 137,2 | 139,6 | 2,4 | 1,7 |
| HB | 759 | 78,2 | 79,5 | 1,3 | 1,6 |  | 33,0 | 34,5 | 1,5 | 4,7 |  | 50,9 | 53,6 | 2,7 | 5,4 |  | 91,2 | 97,2 | 6,0 | 6,6 |
| HH | 1.540 | 57,9 | 61,4 | 3,5 | 5,9 |  | 25,6 | 27,4 | 1,8 | 6,8 |  | 41,2 | 44,2 | 3,0 | 7,3 |  | 80,0 | 86,5 | 6,5 | 8,1 |
| HE | 5.983 | 66,5 | 67,9 | 1,4 | 2,2 |  | 26,3 | 27,6 | 1,3 | 4,9 |  | 41,9 | 44,3 | 2,4 | 5,6 |  | 80,3 | 85,5 | 5,2 | 6,5 |
| MV | 2.256 | 93,1 | 95,2 | 2,1 | 2,2 |  | 38,1 | 39,0 | 0,9 | 2,5 |  | 60,0 | 61,4 | 1,4 | 2,4 |  | 111,6 | 114,3 | 2,7 | 2,3 |
| NI | 9.714 | 83,0 | 85,0 | 2,0 | 2,4 |  | 32,3 | 33,5 | 1,2 | 3,9 |  | 51,6 | 53,9 | 2,3 | 4,4 |  | 99,2 | 104,4 | 5,2 | 5,2 |
| NW | 17.896 | 68,4 | 69,9 | 1,5 | 2,2 |  | 27,1 | 28,1 | 1,0 | 3,8 |  | 43,6 | 45,4 | 1,8 | 4,2 |  | 84,8 | 89,0 | 4,2 | 5,0 |
| RP | 4.717 | 79,9 | 80,5 | 0,6 | 0,8 |  | 30,9 | 31,8 | 0,9 | 3,2 |  | 49,0 | 50,9 | 1,9 | 4,0 |  | 93,0 | 97,8 | 4,8 | 5,2 |
| SL | 1.281 | 86,6 | 88,2 | 1,6 | 1,8 |  | 33,0 | 33,7 | 0,7 | 2,2 |  | 51,9 | 53,2 | 1,3 | 2,6 |  | 96,9 | 100,0 | 3,1 | 3,2 |
| SN | 6.631 | 109,0 | 111,7 | 2,7 | 2,4 |  | 39,8 | 40,7 | 0,9 | 2,1 |  | 63,4 | 64,7 | 1,3 | 2,0 |  | 120,7 | 122,8 | 2,1 | 1,7 |
| ST | 4.195 | 123,8 | 126,1 | 2,3 | 1,9 |  | 46,8 | 47,6 | 0,8 | 1,7 |  | 73,8 | 75,0 | 1,2 | 1,6 |  | 138,1 | 140,3 | 2,2 | 1,6 |
| SH | 2.285 | 54,7 | 55,8 | 1,1 | 2,1 |  | 21,6 | 22,3 | 0,7 | 3,2 |  | 34,0 | 35,3 | 1,3 | 4,0 |  | 63,6 | 66,9 | 3,3 | 5,2 |
| TH | 3.149 | 95,9 | 98,0 | 2,1 | 2,2 |  | 37,6 | 38,3 | 0,7 | 2,0 |  | 59,2 | 60,3 | 1,1 | 1,9 |  | 110,2 | 112,0 | 1,8 | 1,7 |
| Germany | 92.246 | 76,3 | 78,2 | 1,9 | 2,5 |  | 30,6 | 31,8 | 1,2 | 3,8 |  | 48,8 | 50,9 | 2,1 | 4,2 |  | 93,3 | 97,9 | 4,6 | 4,9 |

Legend: F: Rates based on uncorrected population figures; V: Rates based on corrected population data; Abs. diff: Rate difference; Rel. diff.: relative percentage change in rates;

**Suppl. Table 7 Myocardial infarction mortality rates (I21-I22) per 100,000 person-years based on the uncorrected and corrected population figures for 2011-2013, women**

|  |  |  | | | |  | **Age-standardized rates** | | | | | | | | | | | | | |
| --- | --- | --- | --- | --- | --- | --- | --- | --- | --- | --- | --- | --- | --- | --- | --- | --- | --- | --- | --- | --- |
|  | **Deaths** | **Crude rates** | | | |  | **World Standard Population** | | | |  | **European Standarad Population** | | | |  | **Germany 2013 Population** | | | |
|  | **N** | **F** | **V** | **Abs. Diff.** | **Rel. Diff. %** |  | **F** | **V** | **Abs. Diff.** | **Rel. Diff. %** |  | **F** | **V** | **Abs. Diff.** | **Rel. Diff. %** |  | **F** | **V** | **Abs. Diff.** | **Rel. Diff. %** |
| BW | 8.587 | 52,3 | 53,3 | 1,0 | 1,9 |  | 11,5 | 11,9 | 0,4 | 2,9 |  | 20,2 | 20,8 | 0,6 | 3,0 |  | 44,3 | 45,8 | 1,5 | 3,2 |
| BY | 10.394 | 54,1 | 54,4 | 0,3 | 0,7 |  | 12,4 | 12,5 | 0,1 | 0,8 |  | 21,4 | 21,6 | 0,2 | 0,9 |  | 46,6 | 47,1 | 0,5 | 1,0 |
| BE | 2.736 | 50,9 | 53,0 | 2,1 | 4,3 |  | 12,4 | 12,9 | 0,5 | 4,1 |  | 21,7 | 22,6 | 0,9 | 4,0 |  | 47,8 | 49,7 | 1,9 | 4,0 |
| BB | 3.322 | 88,0 | 88,9 | 0,9 | 1,0 |  | 18,4 | 18,6 | 0,2 | 1,2 |  | 32,0 | 32,4 | 0,4 | 1,3 |  | 70,0 | 71,0 | 1,0 | 1,4 |
| HB | 577 | 56,8 | 57,3 | 0,5 | 0,8 |  | 13,0 | 13,2 | 0,2 | 1,5 |  | 21,6 | 21,9 | 0,3 | 1,6 |  | 44,4 | 45,1 | 0,7 | 1,6 |
| HH | 1.353 | 48,9 | 50,7 | 1,8 | 3,6 |  | 11,1 | 11,5 | 0,4 | 3,4 |  | 19,4 | 20,1 | 0,7 | 3,4 |  | 42,6 | 44,0 | 1,4 | 3,3 |
| HE | 4.548 | 48,9 | 49,4 | 0,5 | 1,0 |  | 11,2 | 11,4 | 0,2 | 2,0 |  | 19,2 | 19,6 | 0,4 | 2,1 |  | 41,3 | 42,2 | 0,9 | 2,2 |
| MV | 1.617 | 65,4 | 66,2 | 0,8 | 1,2 |  | 14,3 | 14,5 | 0,2 | 1,5 |  | 24,3 | 24,7 | 0,4 | 1,6 |  | 52,0 | 52,9 | 0,9 | 1,7 |
| NI | 7.451 | 61,8 | 62,6 | 0,8 | 1,2 |  | 13,3 | 13,5 | 0,2 | 1,3 |  | 22,9 | 23,2 | 0,3 | 1,4 |  | 49,7 | 50,4 | 0,7 | 1,5 |
| NW | 15.118 | 55,3 | 55,9 | 0,6 | 1,1 |  | 12,2 | 12,3 | 0,1 | 0,9 |  | 21,1 | 21,3 | 0,2 | 0,8 |  | 45,8 | 46,2 | 0,4 | 0,8 |
| RP | 3.683 | 60,5 | 60,2 | -0,3 | -0,4 |  | 13,1 | 13,2 | 0,1 | 0,6 |  | 22,5 | 22,6 | 0,1 | 0,8 |  | 48,1 | 48,6 | 0,5 | 1,1 |
| SL | 1.037 | 66,7 | 67,5 | 0,8 | 1,2 |  | 13,8 | 14,0 | 0,2 | 1,4 |  | 23,6 | 24,0 | 0,4 | 1,5 |  | 50,2 | 51,0 | 0,8 | 1,7 |
| SN | 5.326 | 84,3 | 85,6 | 1,3 | 1,5 |  | 14,9 | 15,1 | 0,2 | 1,1 |  | 25,9 | 26,2 | 0,3 | 1,1 |  | 56,5 | 57,2 | 0,7 | 1,1 |
| ST | 3.183 | 90,3 | 91,5 | 1,2 | 1,3 |  | 17,8 | 18,1 | 0,3 | 1,3 |  | 30,3 | 30,7 | 0,4 | 1,3 |  | 64,3 | 65,2 | 0,9 | 1,3 |
| SH | 1.782 | 41,1 | 41,2 | 0,1 | 0,4 |  | 9,2 | 9,3 | 0,1 | 1,0 |  | 15,6 | 15,8 | 0,2 | 1,3 |  | 33,1 | 33,6 | 0,5 | 1,6 |
| TH | 2.289 | 68,1 | 69,0 | 0,9 | 1,3 |  | 13,9 | 14,1 | 0,2 | 1,2 |  | 23,8 | 24,1 | 0,3 | 1,2 |  | 50,7 | 51,2 | 0,5 | 1,2 |
| Germany | 73.003 | 58,4 | 59,2 | 0,8 | 1,3 |  | 12,8 | 13,0 | 0,2 | 1,5 |  | 22,1 | 22,4 | 0,3 | 1,5 |  | 47,8 | 48,6 | 0,8 | 1,6 |

Legend: F: Rates based on uncorrected population figures; V: Rates based on corrected population data; Abs. diff: Rate difference; Rel. diff.: relative percentage change in rates;

**Suppl. Figure 1 Relative difference in corrected minus uncorrected mortality rates in two major city states of Germany 2011-2013**

| **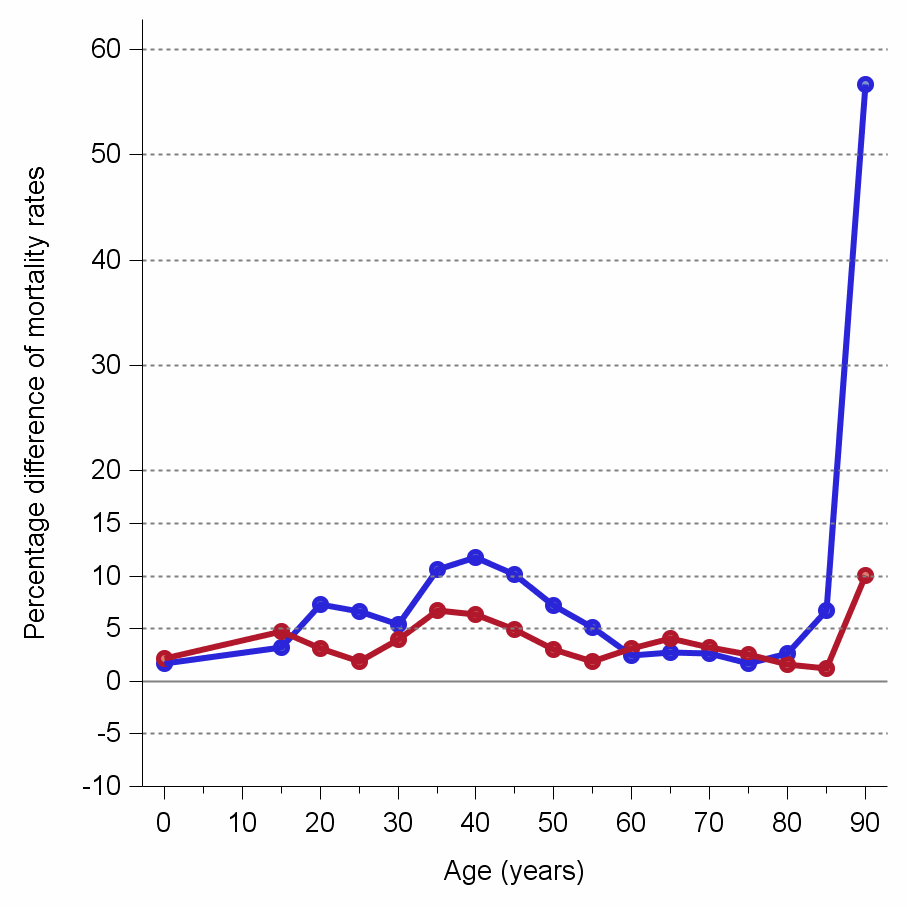** | **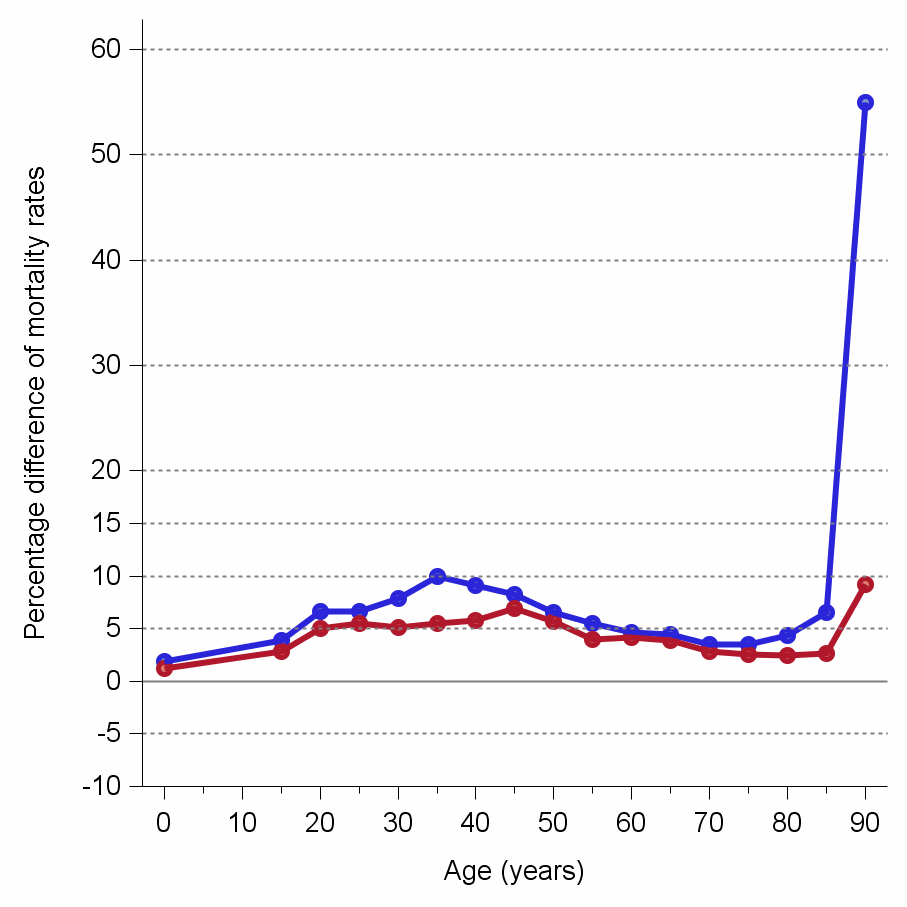** |
| --- | --- |
| Hamburg | Berlin |

**Suppl. Figure 2 Age-mortality patterns of selected cancers in Germany 2011-2013**

| **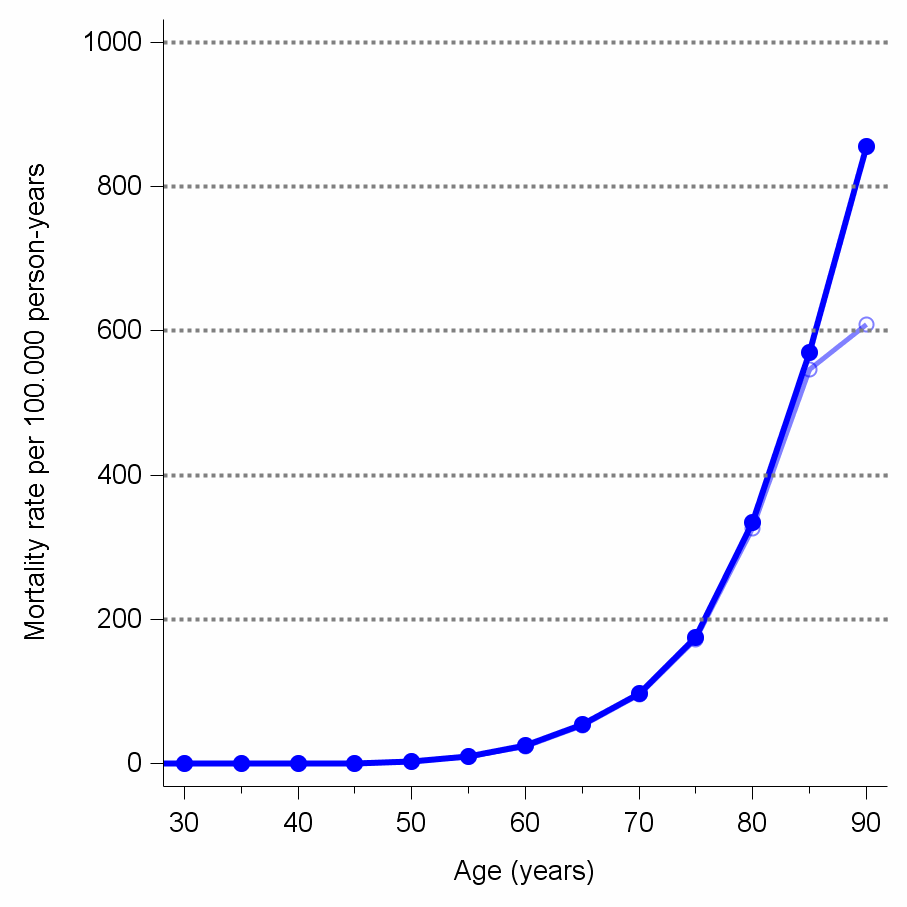** | **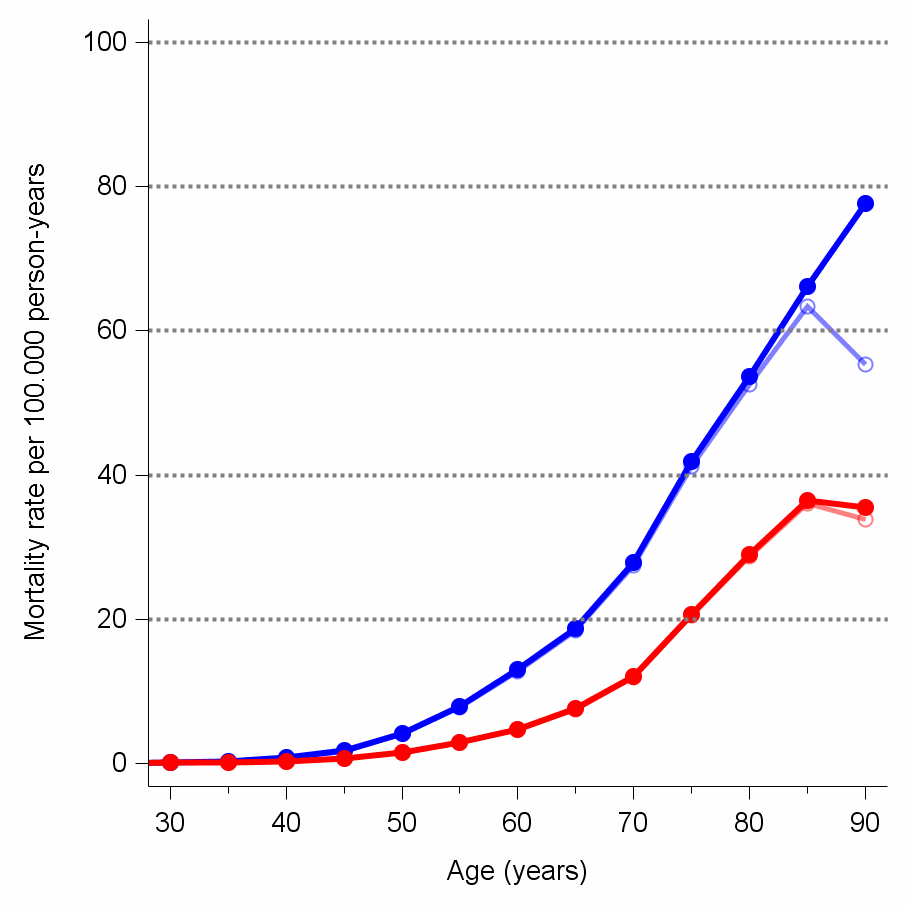** | **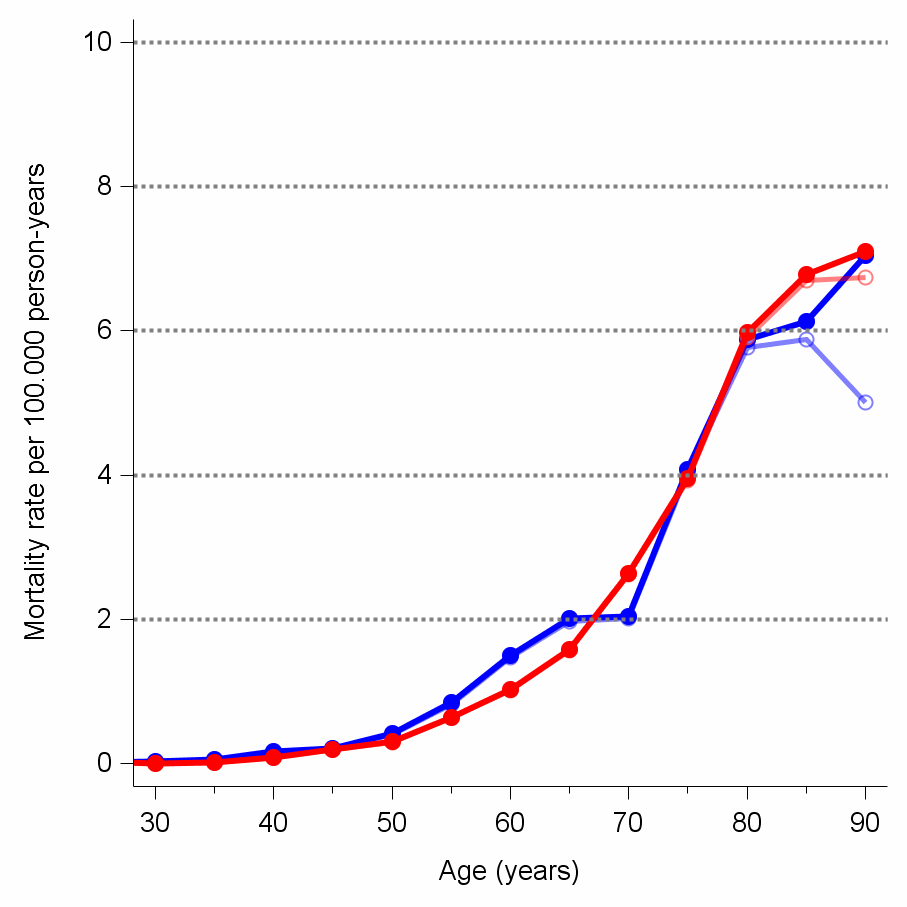** |
| --- | --- | --- |
| **Prostate cancer**  **(ICD-10: C61)** | **Kidney cancer**  **(ICD-10: C64)** | **Thyroid cancer**  **(ICD-10: C73)** |

Legend: rates based on corrected population figures (●), rates based on uncorrected population figures (○); blue: men, red: women; ICD-10: International Classification of Diseases, 10th edition.
